# Data-driven classification of *Escherichia coli* using protein language model ascertains O-serotype determining genes

**DOI:** 10.1038/s41598-026-40783-1

**Published:** 2026-03-19

**Authors:** Heesu Jeong, Hanshin David Shin, Jaehoon Jung, Heebal Kim

**Affiliations:** 1 Interdisciplinary Program in Bioinformatics, Seoul National University, Seoul, 08826 Republic of Korea; 2 Department of Agricultural and Life Sciences and Research Institute of Population Genomics, Seoul National University, Seoul, 08826 Republic of Korea; 3 eGnome, Incorporated, Seoul, 05836 Republic of Korea; 4 Department of Agricultural Biotechnology, Research Institute for Agriculture and Life Sciences, Seoul National University, Seoul, 08826 Republic of Korea

**Keywords:** Machine learning, Microbial genetics

## Abstract

Serotyping of *Escherichia coli* is a critical tool for clinical diagnostics, epidemiology, and vaccine development. Traditional serotyping methods rely on antigen–antibody interactions or reference-based genomic sequence similarity tools that depend on direct matches to predefined serotype gene databases, making their performance highly sensitive to database completeness and limiting their ability to detect or generalize to novel, divergent, or recombinant variants. In this study, we introduce a data-driven approach using a Protein Language Model (PLM) to classify *E. coli* O serotypes. We applied the ESM-2 model to encode protein sequences into vector representations and trained a machine learning classifier to predict serotypes from genomic data. A dataset of 11,272 *E. coli* genomes was analyzed, revealing nine ML-prioritized markers—*wcaM*,* wcaL*,* wcaK*,* wzzE*,* wzxC*,* wecC*,* glmM*,* garR*, and *hisD*—that significantly contribute to O serotype classification. The classification model based on these genes outperformed traditional bioinformatics tools, achieving an accuracy of 93% using a Random Forest classifier. Notably, the proposed model exhibits high recall for low-frequency and underrepresented serotypes, leading to improved balanced performance across classes and contributing to the overall increase in classification accuracy. Our findings suggest that PLM-based analysis enhances serotype prediction, providing a scalable and efficient framework for high-throughput epidemiological surveillance and exploratory bacterial classification, complementing current diagnostic methods.

**Supplementary Information:**

The online version contains supplementary material available at 10.1038/s41598-026-40783-1.

## Introduction

The use of antigen-antibody interactions for bacterial classification is a fundamental aspect of microbiological and immunological research. By leveraging the specificity of these interactions, bacterial species can be classified based on their surface antigens, such as proteins, carbohydrates, or other molecules that elicit immune responses. This technique, known as serotyping, plays a crucial role in various fields, including clinical diagnostics, epidemiology, and vaccine development^1^.

Serotyping is particularly important in the characterization of *Escherichia coli* (*E. coli*), which possesses a wide range of antigenic determinants that define its serological profile. These determinants include lipopolysaccharides (LPS; O antigens), flagellar proteins (H antigens), and capsular polysaccharides (K antigens)^2,3^. The O serogroups, determined by the composition and arrangement of sugars in LPS, are especially diverse, with over 180 recognized O antigen types^4,5^. To identify the O serotype of E. coli, several methods can be employed, such as agglutination assays with specific antisera, PCR-based O-typing, and advanced sequencing technologies.

With the rapid decrease in sequencing costs, bioinformatic tools have emerged as a powerful means for predicting serotypes directly from genomic sequences. Various bioinformatics tools, such as EcOH, ECTyper, and SeroTypeFinder, have been developed to predict O serotypes from genomic sequences^6–8^. These tools use sequence similarity searches to identify the O serotype by comparing genomic sequences against a curated database of genes from the O-Antigen Gene Cluster (O-AGC) and related regions. Among these genes, *wzx* and *wzy* are commonly used as markers, as they encode proteins involved in O-antigen polymerization and export. Given that 93% of O antigens depend on the *wzx*/*wzy* process, these genes are effective for predicting O serotypes^9^. However, more than 80 other genes are present in the O-AGC and contribute to the O-antigen biosynthesis pathway, suggesting that additional genes beyond *wzx* and *wzy* may be critical for accurate serotype prediction^10^. While these bioinformatic tools rely on sequence similarity to identify known serotypes, they can be limited by the completeness of existing databases and may overlook novel or less well-characterized genetic determinants. These limitations motivate approaches that can leverage genome-wide information and capture discriminative signals beyond predefined marker sequences.

Protein language models (PLMs) trained on large-scale protein sequence corpora generate embeddings that capture biochemical and evolutionary constraints from primary sequences. Such embeddings provide general-purpose representations that can be used for downstream prediction tasks, even when explicit annotations are incomplete. Therefore, PLM-based embeddings offer a promising route for robust O-serotype prediction from genomic protein sequences.

Although embedding-based classification has been explored in microbial genomics, prior studies have more commonly demonstrated this paradigm in viral datasets or broad phenotype prediction settings^11,12^. In contrast, embedding-based inference of *Escherichia coli* O-serotypes remains underexplored, despite its clinical and epidemiological importance. Furthermore, beyond experimentally established serotyping markers, systematic data-driven screening to identify additional informative marker genes for O-serotype prediction has been limited.

In this study, we aimed to predict O serotypes in *E. coli* using protein-embedding–based machine learning and to systematically identify informative marker genes beyond canonical loci. With the use of the Microbial Genome Database (MBGD), we conducted an extensive analysis of gene prevalence and classification accuracy across a diverse set of *E. coli* strains^13^. By leveraging ESM-2 PLM, we performed genome-wide screening to identify genes that correlate with specific O serotypes, potentially improving the accuracy of serotype classification and contributing to a deeper understanding of *E.coli* serological diversity^14^. This data-driven approach highlights the potential of PLMs as a complementary framework for large-scale genetic screening, providing an alternative representation-learning perspective alongside established association-based methodologies.

## Results

**Fig. 1:**
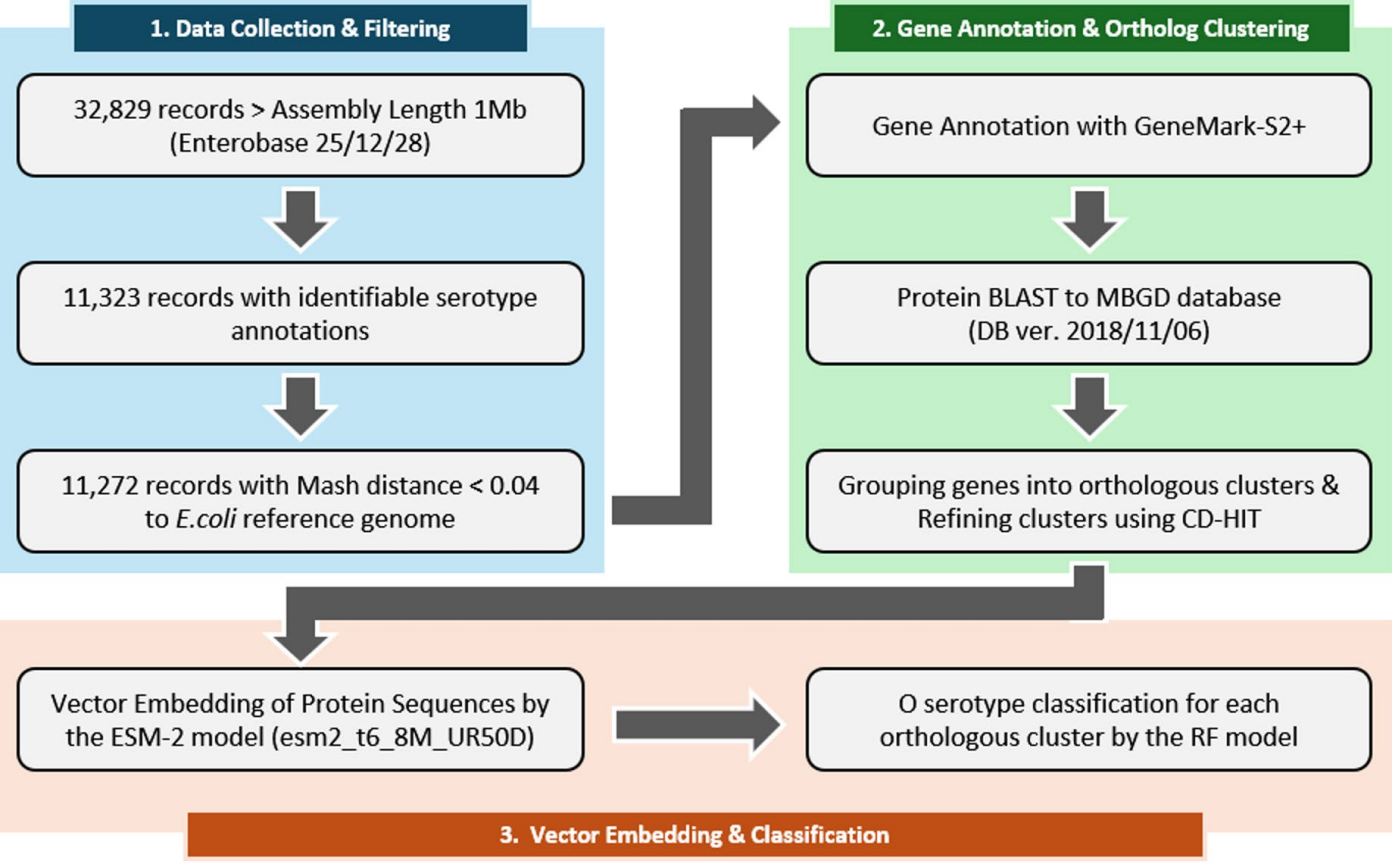
Workflow of the study. *E. coli* genome sequences were collected from Enterobase and filtered based on assembly length and sequence identity with the reference genome. Genes were annotated, and corresponding orthologous gene clusters were assigned according to MBGD BLAST results. Protein sequences from each orthologous gene cluster were converted into fixed-length vectors (length 320) using the ESM-2 model and were used to train the RF classification model.

**Fig. 2:**
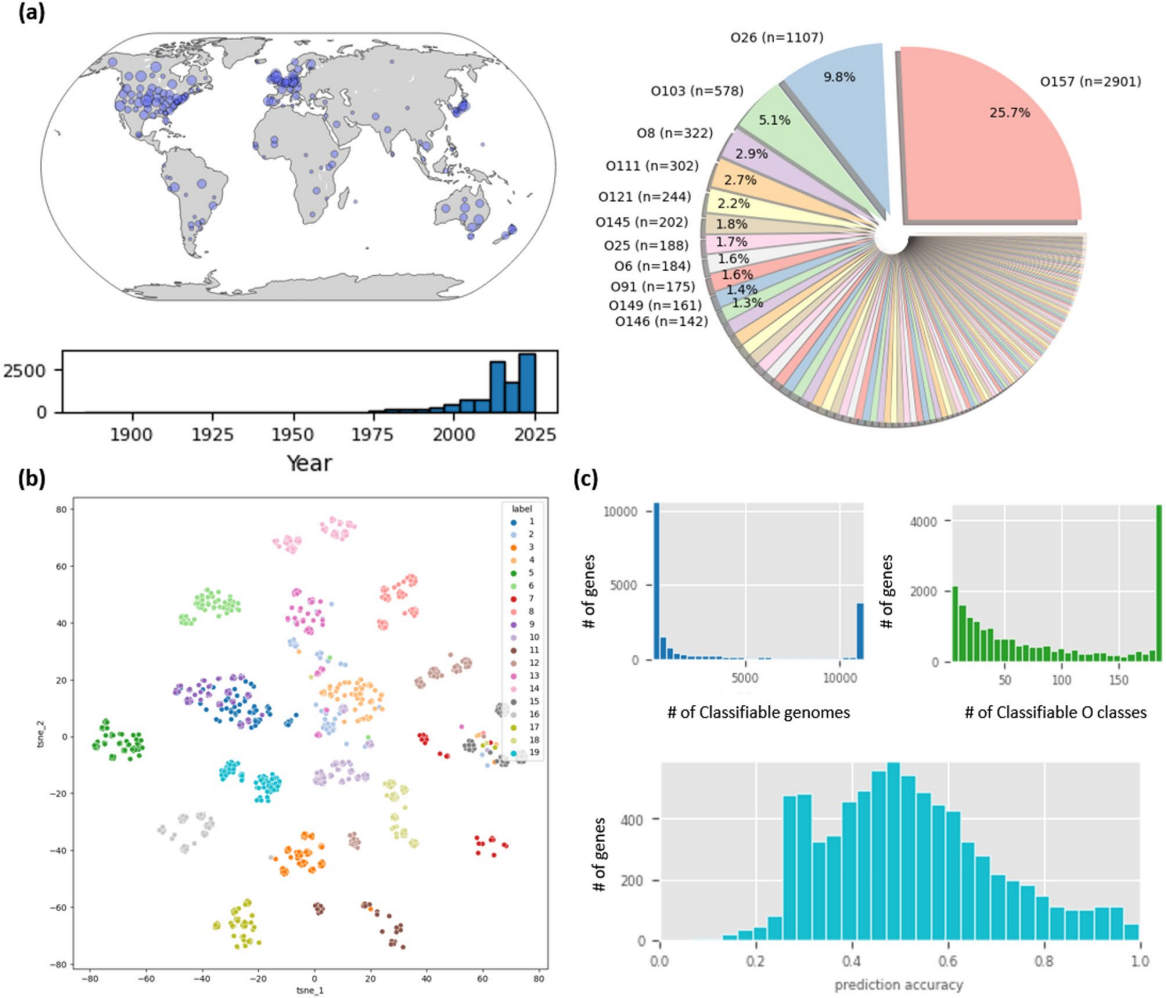
**(a)** Overall statistics of the dataset used in the study, including sampling locations and collection dates (left), and the distribution of O serotypes in the data (right). O157 is the most prevalent serotype, comprising 25.7% of the samples. **(b)** 2D t-SNE plot of embedded vectors using the ESM-2, colored according to protein BLAST results from the MBGD database, representing each orthologous gene cluster. **(c)** Distribution of classification metrics across orthologous gene clusters. The histograms illustrate the performance of individual gene clusters in terms of the total number of genomes successfully classified (upper left), the diversity of O serotypes covered (upper right), and the overall prediction accuracy (bottom).

As of December 28, 2025, a total of 32,829 Escherichia coli genome assemblies were collected from Enterobase, with assembly lengths of 1 Mb or larger (Fig. 1)^15^. This dataset contained 189 distinct O serotypes, with O157 being the most prevalent. Additionally, 161 O serotypes had five or more samples, and 123 O serotypes had ten or more samples, ensuring sufficient representation for building a classification model (Fig. 2a).

Gene annotation of the filtered genomes was conducted using GeneMark-S2+, resulting a total of 58,290,693 annotated genes, averaging 5,171 genes per genome. Orthologous gene clusters were the assigned using the MBGD database (version 2018/11/06) based on protein BLAST best-hit matching^13,16^. MBGD clusters were subsequently merged using CD-HIT to collapse highly similar sequences and produce a non-redundant ortholog set used for downstream feature construction^17^.

We then leveraged ESM-2–based protein embeddings to build an ortholog-aware feature representation for O-serotype classification. Using the ESM-2–derived protein embeddings and ortholog-based gene cluster (hereafter referred to as gene clusters) representations, we first assessed whether the resulting vectors captured meaningful structure among genes and clusters. To this end, we performed 2D t-SNE (t-distributed Stochastic Neighbor Embedding) on representative cluster IDs (1 to 19), which revealed local clustering patterns based on their organization in the learned vector space (Fig. 2b)^18^.

Subsequently, after a stratified 7:3 train–test split of all samples based on O-serotype labels, we trained a Random Forest (RF) classifier for each gene cluster with 10 repeated runs on the training set and evaluated multiclass classification accuracy on the held-out test set. Figure 2c summarizes the distribution of gene cluster–level classification accuracy and the corresponding genomic coverage of each cluster. The distribution of the number of genomes classifiable by each gene cluster shows a distinct bimodal (U-shaped) pattern: approximately 70% of clusters (~ 10,000 clusters) are specific to a small subset (fewer than 2,500 genomes), whereas ~ 20% (~ 3,500 clusters) possess broad classification capability across more than 8,000 genomes. A similar polarizing trend is observed in the number of classifiable O types, where ~ 70% of clusters cover fewer than 150 O types, yet ~ 20% can classify nearly all 180 + O types. In contrast, the prediction accuracy of these clusters follows a near-normal distribution centered around 0.5, with a notable tail extending toward high-accuracy clusters (accuracy > 0.8), indicating that while many genes provide partial signals, a specific subset offers high diagnostic power for O-type determination.

### Identification of ML-prioritized markers

**Table 1:** ML-prioritized markers with High Classification Accuracy for Predicting O Serotype in *E. coli*. The table lists orthologous gene clusters that meet the following criteria: high classification accuracy (≥ 0.75), broad serotype coverage (Serotype Coverage; ≥10 O serotypes), and high prevalence across the dataset (Genome Prevalence; ≥10,000 genomes). For each cluster, the representative gene name, mean classification accuracy with standard deviation (Classification Accuracy), functional annotation (Functional Annotation), total number of strains harboring the gene (Genome Prevalence), the variety of O serotypes it can classify (Serotype Coverage), and the corresponding database identifier (MBGD ID) are provided.

Representative Gene Name	Classification Accuracy (± SD)	Functional Annotation	Genome Prevalence	Serotype Coverage	Ortholog Group ID (MBGD)
*wcaM*	0.88 ± 0.001	Colanic acid biosynthesis protein WcaM	10,786	188	35,854
*wcaL*	0.84 ± 0.001	Glycosyl transferase family 1	10,710	188	19
*wcaK*	0.83 ± 0.002	Colanic acid biosynthesis pyruvyl transferase	10,710	188	2726
*wzzE*	0.84 ± 0.002	LPS O-antigen chain length protein	10,965	185	7478
*hisD*	0.81 ± 0.002	Histidinol dehydrogenase	11,266	189	684
*wzxC*	0.78 ± 0.001	Polysaccharide biosynthesis protein	10,709	188	1911
*wecC* (= *rffD*)	0.78 ± 0.001	UDP-glucose 6-dehydrogenase	11,251	189	201
*glmM* (= *cpsG*)	0.80 ± 0.001	Phosphomannomutase	11,199	188	117
*garR* (= *gndA*)	0.77 ± 0.001	3-hydroxyisobutyrate dehydrogenase	11,260	189	66

Based on the distribution of cluster-level classification accuracy and genomic coverage, we identified a subset of genes that showed both consistently high predictive performance and broad representation across the dataset. Rather than applying rigid predefined cutoffs, we selected genes that jointly exhibited high accuracy (typically ≥ 0.75), involvement in the classification of multiple O serotypes (≥ 10 O types), and presence in a large number of genomes (> 10,000) as ML-prioritized markers to highlight robust and broadly informative candidates (Table 1).

Among the identified genes, *wcaM*, with an accuracy of 0.88 ± 0.001, showed the highest performance in predicting O serotypes. This gene, involved in the biosynthesis of colanic acid, appeared in 10,786 genomes and covered 187 distinct O types, making it a highly reliable marker for O serotype classification. Other ML-prioritized markers, including *wcaL* and *wcaK*, achieved classification accuracies of 0.84 ± 0.001 and accuracy 0.83 ± 0.002), respectively.

Interestingly, *wzzE*, which encodes the LPS O-antigen chain length protein, achieved an accuracy of 0.84 ± 0.002. Other genes, such as *hisD* (accuracy 0.81 ± 0.002), *wzxC* (accuracy 0.78 ± 0.001), and *wecC* (accuracy 0.78 ± 0.001), demonstrated consistent classification performance across a large number of genomes and O types. *hisD*, for instance, was found in 11,266 genomes and covered 188 O types, indicating its broad presence across various *E. coli* strains, despite its primary role in histidine biosynthesis.

The *glmM* gene (= cpsG) was present in 11,199 genomes and covered 187 distinct *E. coli* O serotypes, demonstrating its widespread distribution. In the classification model, *glmM* achieved an accuracy of 0.80 ± 0.001, indicating its substantial contribution to O serotype prediction. Another gene was *garR* (= gndA), which was identified in 11,260 genomes and covered all 188 O serotypes in this study. The classification model showed that *garR* had an accuracy of 0.77 ± 0.001, making it a reliable marker for serotype differentiation.

Collectively, these results highlight the importance of a combination of genes involved in polysaccharide biosynthesis, such as the *wca* genes, and genes responsible for O-antigen chain length regulation and other biosynthetic pathways. These genes are strongly associated with accurate O-serotype classification performance and capture sequence variation informative for serotype differentiation.

### Actual relationship between the ML-prioritized markers and amino acid sequences

**Fig. 3:**
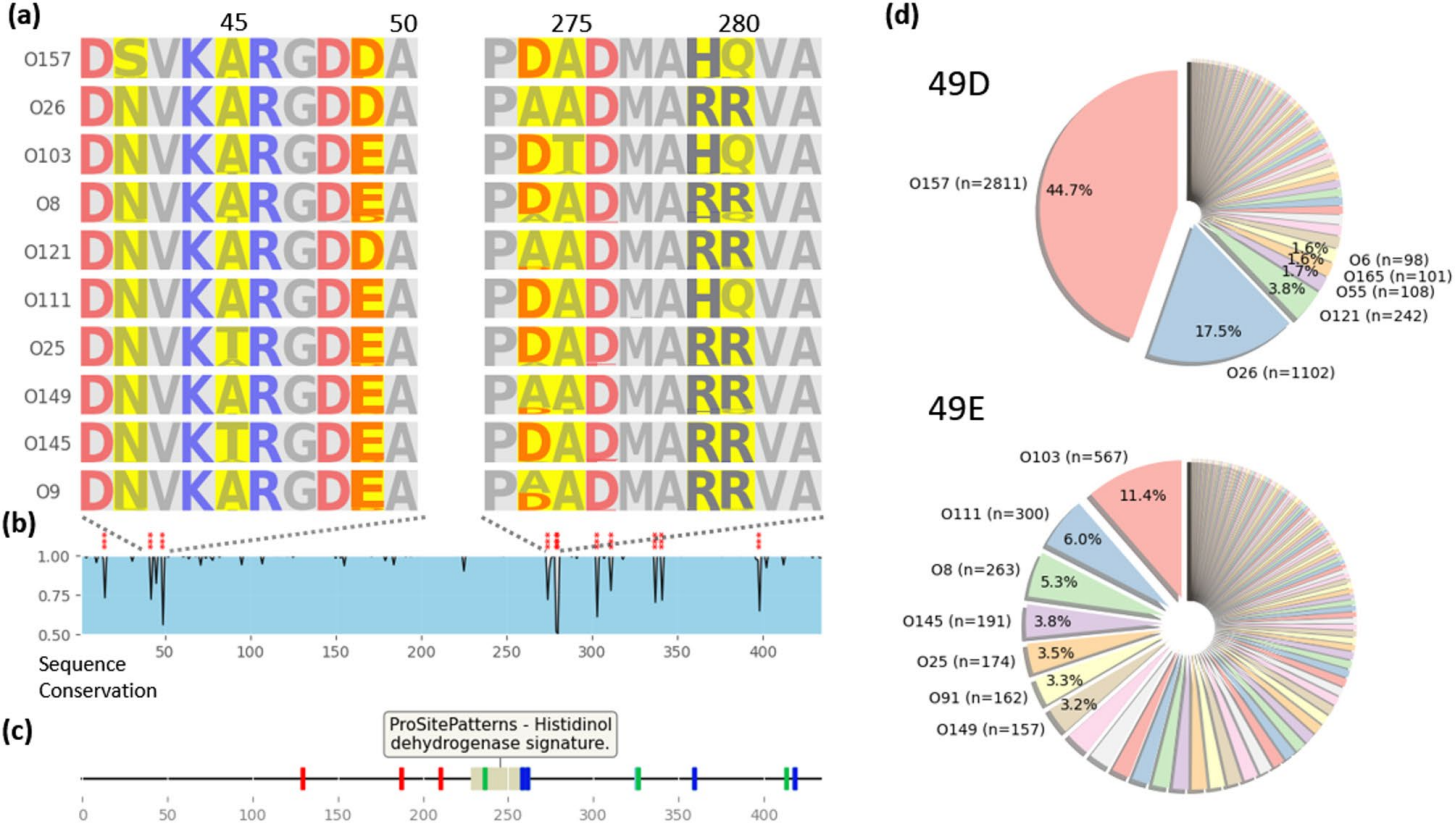
**(a)** Sequence logo of *HisD* protein, highlighting residues 41–50 and 273–282. Residues are color-coded by amino acid charge, with variable regions emphasized. **(b)** Sequence conservation showing the proportion of the most frequent amino acid across the entire sequence. The asterisks indicate positions where sequence conservation falls below 0.8, with statistical significance determined by the Chi-square test (* < 0.05, ** < 0.01, *** < 0.001). **(c)** Features detected by ProSite, designating conserved sites of histidinol dehydrogenase. Additional features annotated in UniProt are color-coded as follows: Red = NAD+ binding site, Green = substrate binding site, Blue = Zn2 + binding site. **(d)** Distribution of O serotypes based on the specific amino acid residue at position 49. The distribution of O serotypes when the 49th residue is Aspartic acid (above) and Glutamic acid (below) is shown.

In the multiple sequence alignment, we identified regions where the proportion of the most frequent amino acid residues at specific positions decreased. These regions were found to correspond to positions that could distinguish different O serotypes. We then analyzed the residue composition ratios by O serotype at these positions, as shown in Fig. 3A and B. This analysis revealed that certain residues were highly conserved across O serotypes, while others showed significant variation.

Notably, in the sequence conservation regions detected by the ProSite program, we found that the differences between O serotypes were smaller compared to those in other regions (Fig. 3C)^19^. This suggests that regions critical to the core functions of the protein tend to be more conserved across all O serotypes, likely reflecting their essential biological roles. In contrast, regions with higher variability may be involved in serotype-specific modifications or interactions.

Additionally, focusing on the HisD protein, we found that when the 49th residue was Aspartic acid (D), the predominant O serotypes included O157, O26, O121, and O55. However, when the residue was Glutamic acid (E), the O serotype distribution shifted to O103, O111, O8, and O145 (Fig. 3D). Although the functional significance of this substitution remains unclear, the consistent serotype-dependent pattern indicates that this residue captures informative sequence variation relevant to O-serotype classification.

### Classification performance of each ML-prioritized marker

**Fig. 4:**
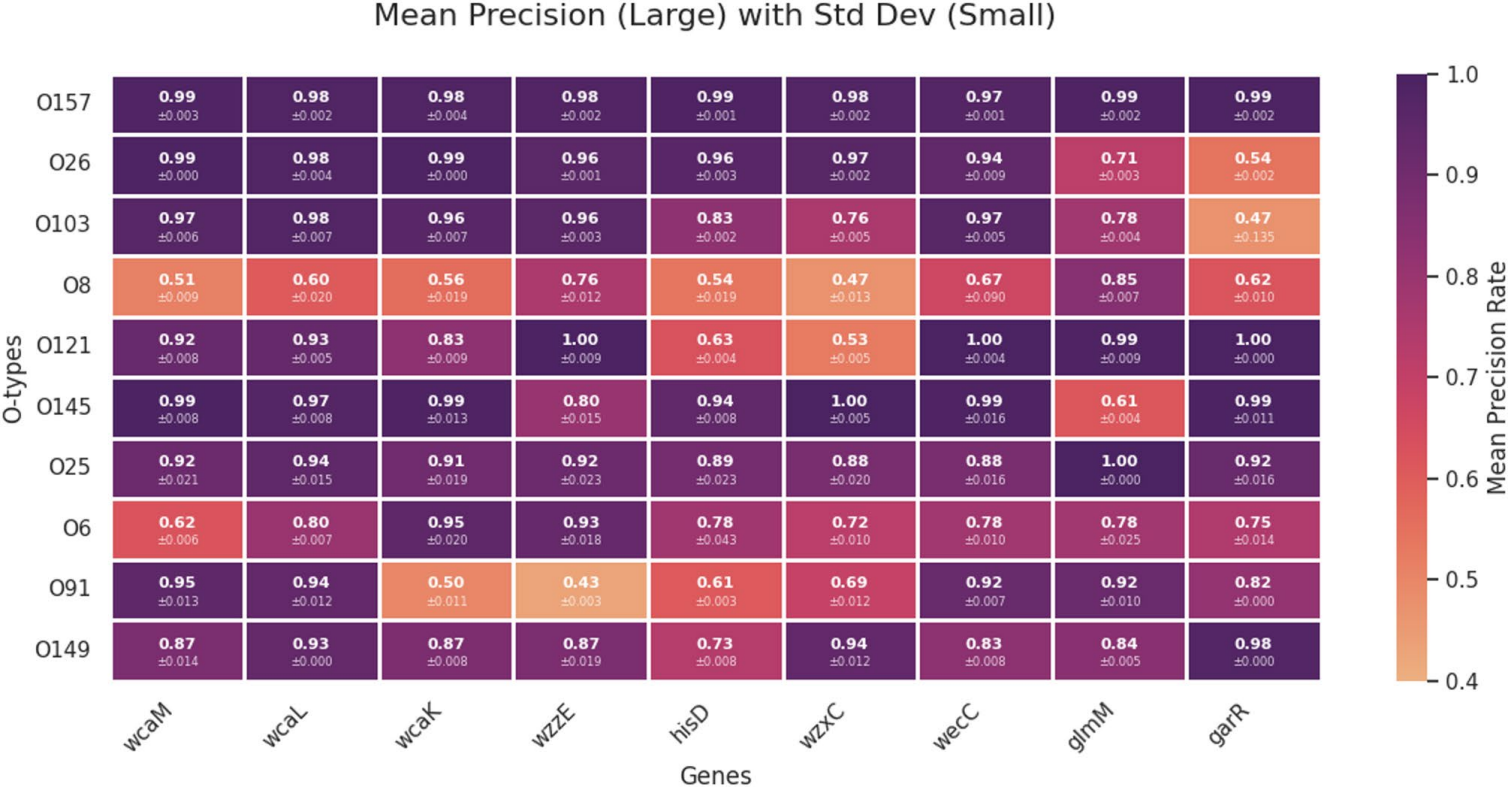
Precision of O-serotype classification based on ML-prioritized markers. The heatmap displays the mean precision rates (large numbers) and their standard deviations (small numbers) for the 10 most prevalent O serotypes, evaluated using representation vectors derived from each ML-prioritized marker. The color gradient represents the Mean Precision Rate, ranging from 0.4 (light orange) to 1.0 (dark purple).

Following this analysis, we evaluated the classification performance for each O serotype specifically to examine how individual genes contribute to serotype identification. We assessed the precision of classification models built on each gene vector across the major O serotypes. The results showed that classification performance varied significantly depending on the gene and serotype, suggesting a strong potential for mutual complementarity (Fig. 4).

For serotypes with sufficient data, such as O157, all tested genes demonstrated uniformly high precision, mostly exceeding 0.97. However, for other serotypes, performance fluctuations were more pronounced. Notably, while the *wzxC* gene is a widely used classification marker, it exhibited relatively lower precision for O103 (0.76), O8 (0.47), O121 (0.53), and O6 (0.72). In these instances, markers like *garR* (e.g., 1.00 for O121) or *wecC* (e.g., 0.97 for O103) showed superior performance, effectively compensating for the limitations of *wzxC*.

Interestingly, the complementarity was not limited to a single gene. For the O8 serotype, where most genes showed sub-optimal performance, *glmM* (0.85) and *wzzE* (0.76) emerged as the most reliable markers. Conversely, for the O91 serotype, *wcaM* (0.95) and *wcaL* (0.94) showed high precision, while *wcaK* (0.50) and *wzzE* (0.43) performed poorly. These findings underscore that a multi-marker approach, leveraging the specific strengths of different genes, is essential for robust O-serotype classification across a diverse range of types.

### Performance metrics of the classification model using all nine ML-prioritized markers

**Fig. 5:**
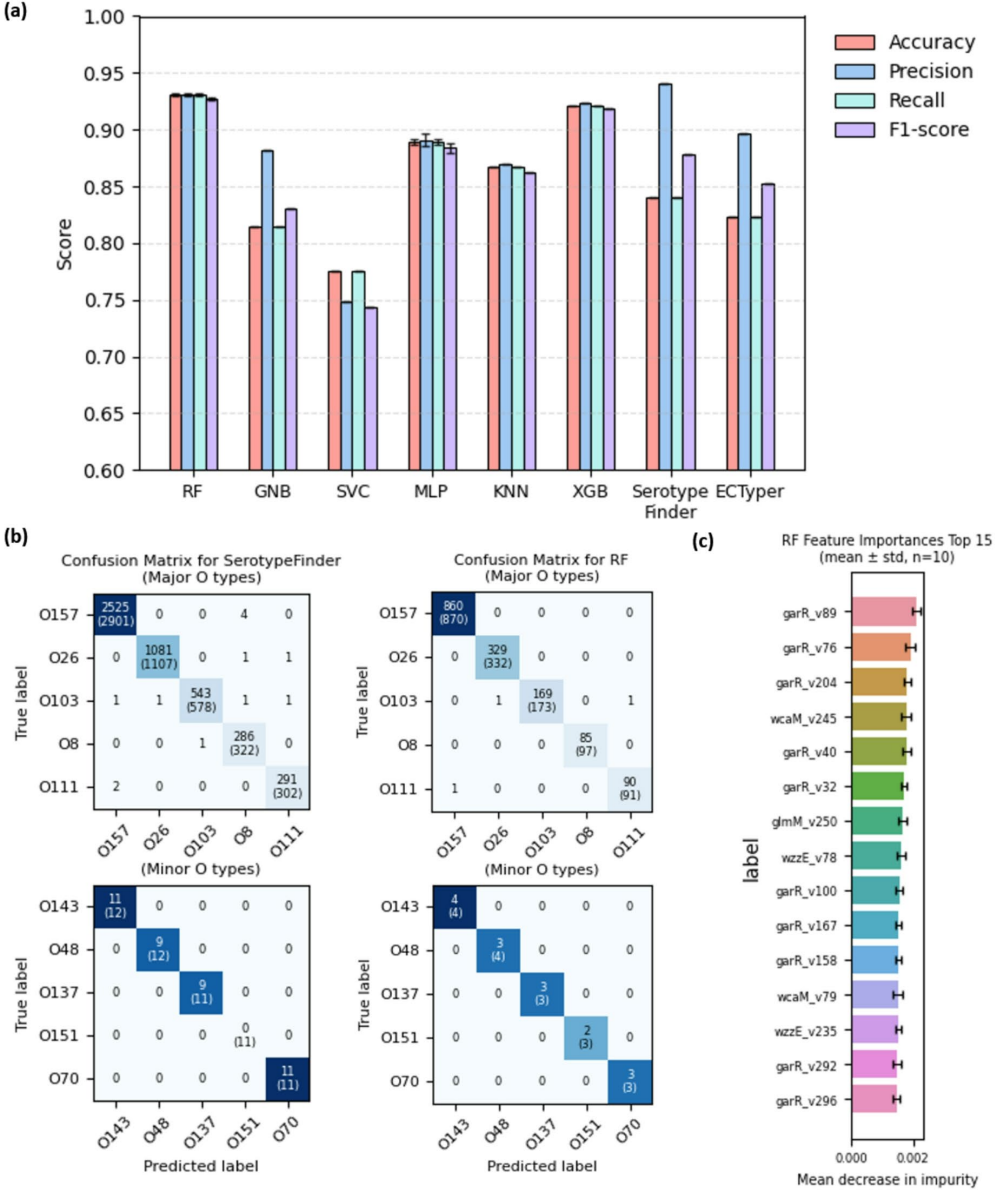
Benchmarking of O-serotype classification models using ML-prioritized markers against canonical tools. **(a)** Performance comparison across various classification models. Six machine learning (ML) models trained on the nine ML-prioritized markers were compared with standard tools (SerotypeFinder, ECTyper). RF = Random Forest, XGB = XGBoost, GNB = Gaussian Naïve Bayes, MLP = Multilayer Perceptron, SVC = Support Vector Classifier, KNN = K-Nearest Neighbor. Evaluation metrics include Accuracy, Precision, Recall, and F1-score. For ML models, error bars represent the standard deviation derived from 10 independent runs. **(b)** Confusion matrices for SerotypeFinder and the RF model. The top 5 most frequent serotypes are categorized as ‘Major O types’, while the 120th to 124th most frequent are categorized as ‘Minor O types’. The total number of genomes for each true label is provided in parentheses. **(c)** Feature importance in the RF model. The top 15 features (dimensions) from the nine ML-prioritized markers are ranked by their mean decrease in impurity. Error bars indicate the standard deviation calculated from 10 independent runs.

To assess the performance differences compared to existing programs, we measured the performance of each machine learning (ML) models trained on the nine ML-prioritized markers against canonical serotype prediction programs. The ML models included ensemble tree-based algorithms like Random Forest (RF) and XGBoost, neural network-based Multilayer Perceptron (MLP), Support Vector Classifier (SVC) using a linear kernel, as well as Gaussian Naive Bayes (GNB) and K-Nearest Neighbor (KNN) classifiers. Performance metrics were averaged over 10 independent runs to ensure robustness.

The tree-based models exhibited the highest overall performance; the RF model achieved a mean accuracy of 0.93 (± 0.005), followed by XGBoost with 0.92 (± 0.004) (Fig. 5a). The MLP model followed with an accuracy of 0.89 (± 0.002), while KNN (0.87 ± 0.001), GNB (0.81 ± 0.001), and SVC (0.77 ± 0.001) showed lower performance levels. The relatively lower performance of MLP may be attributed to the high parameter space relative to the available training data.

To further validate classifier stability, we analyzed micro-average ROC and Precision-Recall (PR) curves (Fig S2). Consistent with the accuracy metrics, tree-based ensembles demonstrated superior discriminative power (ROC-AUC > 0.98) and robust precision–recall trade-offs (PR-AUC > 0.94) even under class imbalance. In contrast, SVC and GNB exhibited marked declines in precision at higher recall levels, confirming the reliability of tree-based models for this high-dimensional task.

For existing canonical programs, SerotypeFinder showed an average performance of 0.84 and ECTyper averaged around 0.82, indicating that they performed less well than the decision tree-based models. However, when considering precision as a metric, both canonical programs exhibited very high performance, surpassing the ML-based classifiers. When using the F1-score—which takes both precision and recall into account—the decision tree-based models still outperformed the canonical tools, similar to the evaluation based on accuracy, while the other ML models lagged behind.

To examine the classification results in more detail, we analyzed the outcomes from SerotypeFinder and the RF model in the form of confusion matrices, focusing on the five most prevalent major O types and the minor O types ranked from 120th to 124th in frequency (Fig. 5b). The results showed that for the major O types (O157, O26, O103, O8, O111), both SerotypeFinder and the RF model correctly classified a substantial number of genomes, achieving accuracies of 90.7% (4,726/5,210) and 98.1% (1,533/1,563), respectively.

However, for the minor O types (O143, O48, O137, O151, O70), different trends were observed between the two methods. SerotypeFinder performed well on O70 (100%) and O143 (91.7%), but completely failed to classify the O151 type, resulting in 0% (0/11) accuracy. In contrast, the RF model demonstrated more robust coverage across rare types, successfully identifying O151 with 66.7% (2/3) accuracy and achieving 100% accuracy for O143, O137, and O70.

To further investigate how much these ML-prioritized markers contribute to the overall classification model, we constructed a Random Forest model and examined the most important features for overall performance (Fig. 5c). As a result, variants of the *garR* gene (such as garR_v89 and garR_v76) were identified as the most statistically most informative features in the overall classification. Other genes, including *wcaM*, *glmM*, and *wzzE*, were also observed to significantly influence the model’s ability to distinguish between different serotypes.

## Discussion

In this study, we successfully showed the potential of Protein Language Models (PLMs) for predicting *E. coli* O serotypes based on genomic data. By utilizing vector representations of protein sequences, we identified ML-prioritized markers beyond the traditional *wzx* and *wzy* markers that correlate with specific O serotypes. These findings not only enhance our understanding of *E. coli* serotype diversity but also provide a more comprehensive framework for serotype classification compared to existing methods relying solely on sequence similarity searches.

This approach represents a significant shift from heuristic and experimental genomic studies to a more structured, data-driven methodology. The application of PLMs in our research enabled the transformation of protein sequences into meaningful 320-dimensional vector representations. Despite the computational limitations necessitating the use of the smallest possible vector embeddings, these embeddings successfully encapsulated essential information, reflecting unique properties of each gene’s protein sequence. This capability was further validated through 2D t-SNE visualizations, which revealed that the embedded gene vectors exhibited clustering patterns corresponding to their evolutionary and functional units, as defined by MBGD orthologous gene clusters.

### Biological interpretation of ML-prioritized marker genes

Distinguishing among nearly 180 O serotypes requires substantial heuristic knowledge and academic effort. Encouragingly, the genes predicted by our model to be associated with O serotypes largely overlapped with the O-Antigen Gene Cluster (O-AGC), reinforcing their possible role in serotype determination. Specifically, the nine ML-prioritized markers (*wcaM*,* wcaL*,* wcaK*,* wzzE*,* wzxC*,* wecC*,* glmM*,* garR*, and *hisD*) identified in this study include genes frequently mentioned in relation to the O-AGC. These genes include both canonical O-antigen–related loci and additional genes whose functional links to O-antigen or lipopolysaccharide biosynthesis are not yet fully established but show consistent associations with serotype variation.

*wcaL* and *wcaM* are involved in the colanic acid biosynthesis pathway, which is essential for the synthesis of capsular polysaccharides (CAPs). WcaL encodes a glycosyltransferase that plays a crucial role in adding sugar residues to the colanic acid structure and is essential for colanic acid biosynthesis in the *E. coli* K-12 strain^20^. The role of WcaM in capsular polysaccharide (CAPs) synthesis remains unclear. However, *wcaM* is annotated as a colanic acid biosynthesis gene, and mutations in *Salmonella* have been associated with reduced biofilm formation, suggesting a possible involvement in cell-surface polysaccharide–related processes^21^. Given the close evolutionary relationship between *Salmonella* and *E. coli*, these observations provide indirect contextual support for interpreting WcaM in the context of *E. coli* serotype determination and CAPs biology.

Additionally, *wcaK*, another gene involved in colanic acid biosynthesis, is believed to encode a pyruvyl transferase, with the addition of a pyruvyl group to the terminal galactosyl residue of the E-unit side chain proposed as a step in the pathway^20^. Importantly, biochemical evidence supports the formation of the final glycosylated hexasaccharide product by WcaL, further highlighting the role of these enzymes in colanic acid modification^22^.

*wzzE* encodes a protein that regulates the length of the O-antigen polysaccharide chain^23,24^. The length of the O-antigen chain is a critical determinant of serotype variation, as different lengths of the repeating units contribute to distinct O serotype profiles^9^. WzzE plays a significant role in controlling the polymerization process, and its importance in serotype determination is highlighted by its high classification performance. *wzxC* encodes an O-antigen flippase, which transports lipid-linked O-antigen repeat units across the inner membrane into the periplasm^25^. This step is essential for O-antigen biosynthesis, and *wzxC* has been identified in various *E. coli* strains as a key component of the O-antigen assembly pathway^9,26^. *hisD* encodes histidinol dehydrogenase, a key enzyme in histidine biosynthesis. Although *hisD* is primarily involved in amino acid metabolism, its proximity to the O-antigen biosynthesis gene cluster in *E. coli* may reflect an association with serotype-specific genomic contexts, potentially arising from genetic linkage or co-regulation, rather than a direct functional role in O-antigen biosynthesis^26^.

*wecC* (also known as *rffD*) encodes UDP-N-acetylglucosamine 6-dehydrogenase and plays a crucial role in the biosynthesis of enterobacterial common antigen (ECA), which have interdependence with O Antigen pathways^27,28^. The *rff* operon, which includes *rffD*, along with *rfe*, plays a crucial role in the synthesis of O-antigen precursors. These genes are involved in the production of GlcNAc-pyrophosphorylundecaprenol (lipid I), the first lipid-linked intermediate in ECA synthesis, which is also related to the synthesis of lipopolysaccharide (LPS) O-side chains^29^. *glmM* (also known as *cpsG*) encodes a phosphomannomutase involved which is responsible for CA synthesis, as a gene of the capsule GDP-fucose pathway^30–32^. As part of the *cps* operon regulated by the RcsA/RcsB network, *glmM* may influence O-antigen expression due to its role in colanic acid biosynthesis and its chromosomal proximity to *rfb* genes involved in lipopolysaccharide assembly, suggesting potential regulatory or functional interactions between capsule and O-antigen synthesis pathways^33^. *garR’s* direct involvement in O serotype determination is not fully understood, but its ortholog, *gndA*, is known to define the boundaries of the O-antigen gene cluster (O-AGC)^34^. The high classification accuracy in our study suggests that *garR* may play an indirect role in serotype prediction, potentially reflecting its genomic linkage to the O-antigen gene cluster rather than a direct influence on O-antigen structure or synthesis.

### Comparison of ML models with canonical prediction tools

The serotype prediction model using these ML-prioritized markers also allowed for an assessment of the predictive performance of each gene. In contrast to previous reports, the *wzxC* gene exhibited lower classification performance in our evaluation. This discrepancy can be attributed to the fact that the traditional definition of the *wzxC* gene encompasses two distinct gene clusters. Canonical classification tools, such as SerotypeFinder (*wzx/wzy/wzm/wzt*), ECTyper (*wzx/wzy*), and SRST2 (*wzx/wzy/wzt/wzm*), utilize fixed pools of genes as markers for classification. However, our study revealed that the *wzx* gene exhibited lower classification performance compared to other genes. This issue arose specifically because the range of *wzx* gene clusters employed in this study differed from those used in existing classification tools.

For example, in the case of the O111 serotype, we observed instances where the *wzxC* gene was not detected. Upon further investigation, it was found that the *wzxC* gene identified by tools like SerotypeFinder corresponds to the sequence of MBGD Cluster 5807. This cluster is distinct from MBGD Cluster 1911, which was identified in our study as corresponding to a different amino acid sequence but shares the same *wzxC* designation. Furthermore, MBGD Cluster 5807 represents a smaller orthologous gene set, found in approximately 1,000 genomes, highlighting the difference in classification criteria between our approach and canonical tools. This shows that using the globally defined orthologous gene set from MBGD ensures compatibility with gene sets defined from a broader perspective of bacterial species. This approach would allow for more precise identification of similar serotypes in closely related species and provide a detailed assessment of how each gene influences serotype determination.

While traditional programs (SerotypeFinder, ECTyper) showed higher precision compared to ML-based O serotype classification, they exhibited lower recall rate. This suggests that traditional programs, which rely on highly curated datasets, achieve high specificity but may lack generalizability (sensitivity) across the entire dataset. A similar trend was observed with the ECTyper program. Our ML model showed strong performance across the full dataset, including areas where traditional programs might fail, as reflected in a superior F1 score. This pattern was also observed through the confusion matrix, where both SerotypeFinder and our ML model had high recall rates for the major serotypes. However, differences were seen for minor serotypes, where traditional tools either performed well or failed, while the ML model showed some ability to classify O types that were not covered by the canonical tool. This indicates that traditional canonical tools, which rely on curated databases, can accurately classify specific types registered in the database, securing high precision. However, these tools have limitations in extending to new datasets that are not included or updated in the database, which can be overcome by using ML models.

### Benefits and limitations of using PLM-based approach for genetic studies

In this study, genes previously identified as part of the O-Antigen Gene Cluster were also prioritized by our analysis as informative markers associated with O-serotype classification. This suggests that prediction models based on PLM and data-driven approaches can serve as a valuable tool in forward genetics. A prominent example of genome data-based forward genetic approaches is SNP-based Genome-Wide Association Studies (GWAS). These studies often assume bi-allelic features (Reference allele / Alternative allele) for each locus, resulting in 2 (Additive / Dominant / Recessive models) or 3 categories (Categorical model) per diploid genome, which are then statistically modeled against a response variable.

However, the statistical modeling in these methods has limitations. The influence of each individual SNP locus on overall protein variation can be minimal, and if the functional changes or differences in the entire protein result from non-linear combinations of these loci, the explanatory power of such models becomes restricted. To overcome this, alternatives such as analyzing haplotypes, which include multiple loci simultaneously, or adopting multi-omics approaches for broader explorations, have been proposed.

In contrast, this study highlights the advantage of using vector embedding to represent the differences in entire proteins as features, particularly when utilizing PLMs. Despite the complexity of the task—relating 320-dimensional feature representations to over 100 O-serotypes—the model showed measurable predictive performance across classes and prioritized informative features associated with classification outcomes. This underscores the effectiveness of this approach, even in scenarios far exceeding the typical number of independent and response variables in prior studies.

Additionally, one advantage of converting specific genes into sequence-based vectors is that it enables various numerical operations based on digitized values. This is particularly beneficial when there are multiple orthologous groups within a single genome that perform similar functions or can be grouped as paralogs. By calculating the average of these values, a representative value for each group can be obtained, which can then be utilized in model construction and prediction.

The successful identification of associated genes based on genomic data in this study is encouraging. However, one limitation of this study is that the application of PLM required relatively significant GPU computational resources. This limitation may be addressed over time as smaller and optimized models, such as Small Language Models (SLMs) or on-device LLMs, are emerging. These developments suggest that similarly compact PLM models could be adapted for use on standard laboratory-level computers in the future.

While our embedding-based framework shows strong predictive potential, certain limitations merit further discussion. First, the reliance on MBGD orthologous clustering means that any inherent inconsistencies in ortholog assignment could propagate to the gene-level representations. Since ortholog definitions are subject to database-specific criteria and versioning, the stability of our marker prioritization should be interpreted within the context of the chosen annotation framework.

Additionally, despite our efforts to address class imbalance through stratified sampling, the predictive power for rare O-serotypes remains constrained by limited sample sizes. The generalizability of the model to these infrequent types requires broader validation using more expansive, real-world surveillance data.

Another limitation lies in the assumption that the effects of each gene are independent when identifying higher-order genes influencing the O serotype. This assumption excludes the possibility of epistasis, where multiple genes interact in a complex manner to determine the O serotype. This limitation arises from the fact that in this study, each gene corresponds to a 320-dimensional vector, and developing a model that simultaneously considers the entire orthologous group would require optimizing parameters for 245,328,320 input variables (766,651 × 320).

Lastly, the ML-prioritized markers should be viewed as high-confidence candidates rather than experimentally confirmed determinants. The associations captured by our protein embeddings may partly reflect genomic linkage or lineage-specific backgrounds. Reflecting this distinction, the current framework is best positioned for high-throughput epidemiological surveillance and exploratory research rather than immediate clinical diagnosis. While it offers a powerful tool for screening large genomic datasets, its application as a primary diagnostic method requires further prospective validation against standard biochemical assays. Consequently, future experimental assays are essential to move beyond statistical association and establish the mechanistic roles of these genes in defining O-serotypes.

Despite this, the list of genes obtained in this study successfully includes those genuinely related to O serotype determination. This success is attributed to the study’s focus on bacterial species, where genetic variations are more directly linked to phenotypic variance. In contrast, organisms such as eukaryotes and multicellular species, which exhibit diverse gene regulation mechanisms—such as expression regulation through epigenetics, splicing, and other transcriptional processes—present greater challenges for extracting phenotype-associated gene lists using this approach. Overcoming these challenges will likely require additional methodological advancements tailored to these more complex systems.

Building on this foundation, our results are consistent with previous studies that highlight the importance of the O-antigen gene cluster (O-AGC) in O serotype determination. However, the use of PLMs allowed us to go beyond conventional approaches by identifying additional genes within the O-AGC that play a significant role in O-antigen biosynthesis. This suggests the utility of machine learning-driven approaches in advancing the field of bacterial serotyping, offering a data-driven framework that not only predicts serotypes but also identifies genomic features consistent with the biological underpinnings of E. coli serotypes.

## Matherials & methods

### Data collection & preprocessing

All data used in this analysis were collected from 32,829 records classified under E. coli & Shigella in Enterobase (as of December 28, 2025), restricted to assemblies annotated as E. coli and with assembly lengths ≥ 1 Mb. Assemblies were further filtered for unambiguous serotype annotations (a single O type and H type), and genomic similarity to the E. coli reference genome ASM584v2 (GCA_000005845) was assessed using MASH^35^. A MASH distance threshold of < 0.04, corresponding to an ANI > 0.96, which is a commonly used cutoff in microbial genomics to ensure accurate species-level classification, was applied^36,37^. These filtering steps resulted in the exclusion of 51 assemblies and yielded a final dataset of 11,272 genomes.

### Gene annotation & orthologous group assignment

Protein-coding genes were predicted from each genome using GeneMark-S2 + with the genome type set to “bacteria.” The resulting protein sequences were queried against the MBGD protein database (version 2018/11/06) using BLASTP (e-value ≤ 1e − 5, max-target-seqs = 1, max-hsps = 1), and the best hit based on the lowest e-value was selected. Each protein was assigned to the corresponding MBGD orthologous gene cluster based on this best match, enabling consistent ortholog-based representation across genomes. When a single protein sequence matched multiple MBGD clusters, it was assigned to all corresponding clusters. Subsequently, to examine and separate the heterogeneity among the gene sequences corresponding to a single MBGD cluster, CD-HIT was performed with the parameters -c 0.50 -n 2 -d 0 -T 24 -M 300,000, and the groups that were separated in the results were ultimately designated as distinct orthologous groups^17^.

### Vector embedding using ESM-2 protein language model

Protein sequences corresponding to orthologous gene clusters were converted into fixed-length numerical representations using the pretrained ESM-2 protein language model (esm2_t6_8M_UR50D). Each protein sequence was embedded into a 320-dimensional vector using the esm2 package and the esm-extract script. The parameter --toks_per_batch was set to 4096, num_layer was set to the default value of − 1, include was set to “mean” to obtain the average representation across the entire sequence, and --truncate_seq_length was set to the default value of 1022. The embedding process was performed on two NVIDIA V100 GPUs and used for downstream feature construction and model training. Dimensionality reduction for visualization of embedding space was performed using t-SNE with default parameters (n_components = 2, perplexity = 30.0, learning_rate="auto”, max_iter = 1000, init="pca”) implemented in the Python scikit-learn package and visualized with matplotlib^38,39^.

### Training classification model using the embedded vectors

To evaluate the performance of these models, we employed a fixed stratified train-test split strategy (7:3 ratio) using the StratifiedShuffleSplit algorithm from scikit-learn^38^. This approach ensured that the class proportions of O-serotypes remained consistent across both training and testing datasets, providing a more robust evaluation under the data imbalance. Gene clusters with fewer than three O serotype class labels and fewer than ten data records were excluded from the Random Forest (RF) model construction. In cases where multiple gene vectors derived from common orthologous gene clusters were present within a single genome, the average of these vector values was used.

To prioritize informative gene candidates for downstream modeling, we applied a set of predefined screening criteria based on classification performance and dataset coverage. Specifically, genes were retained if they achieved a multiclass classification accuracy of > = 0.75, were associated with at least 10 different O-serotypes, and were present in at least 10,000 genomes. These thresholds were established as pragmatic and exploratory filters rather than statistically optimized cut-offs, aimed at identifying a tractable subset of high-confidence markers with broad representation across the species. The accuracy threshold (0.75) was selected to capture gene clusters in the upper-performance range of the observed accuracy distribution, which was centered around approximately 0.5 across clusters. The serotype and genome coverage criteria were introduced to ensure that selected markers were broadly represented across the dataset and not primarily driven by performance within narrowly distributed or low-frequency serotype groups. Final classification decisions were determined based on the highest predicted probability among classes (argmax rule), without applying an additional confidence threshold.

For the integrated model using the nine genes, if an orthologous gene cluster was not observed in a given sample, its vector value was padded with 0.0. To confirm that the padding strategy did not bias model performance, we compared zero-padding with alternative values (1 and − 1). Test accuracy and feature importance rankings were highly consistent across all settings, indicating that the choice of padding value had minimal impact on downstream analyses. To ensure the reproducibility of our machine learning models, we specified precise hyperparameters for each algorithm. All models were implemented using the scikit-learn^38^ and XGBoost^40^ libraries in Python. We evaluated multiple supervised learning algorithms using protein embedding vectors as input features, including Random Forest (RF), Support Vector Classifier (SVC), Gaussian Naïve Bayes (GNB), Multilayer Perceptron (MLP), k-Nearest Neighbors (KNN), and XGBoost (XGB). To ensure consistency and reproducibility across gene-level comparisons, fixed hyperparameter settings were used for all models unless otherwise specified.

The Random Forest classifier was trained with 100 decision trees (n_estimators = 100), using entropy as the splitting criterion (criterion=’entropy’) and a maximum tree depth of 10 (max_depth = 10), with other parameters kept at default. The Support Vector Classifier (SVC) employed a linear kernel (kernel=’linear’) with all remaining hyperparameters set to their default values (e.g., C = 1.0). The Multilayer Perceptron (MLP) consisted of a single hidden layer with 500 units (hidden_layer_sizes=(500,)), utilizing the Rectified Linear Unit activation function (activation=’relu’) and the Adam solver (solver=’adam’); all other parameters were maintained at default settings. For gradient boosting, XGBoost models were configured with a maximum tree depth of 10 (max_depth = 10) and a learning rate of 0.1 (eta = 0.1). Gaussian Naïve Bayes (GNB) and k-Nearest Neighbors (KNN) classifiers were trained using standard default settings; specifically, KNN utilized 5 neighbors (n_neighbors = 5) with uniform weighting (weights=’uniform’) while leveraging parallel computation (n_jobs = 40). To ensure reproducibility and assess model stability across the 10 independent runs, random seeds were systematically assigned by incrementing a base seed of 33 by 1 for each iteration (i.e., seeds 33 to 42).

For performance comparison with the developed ML models, we employed widely used canonical serotyping tools. Specifically, benchmarking was conducted using SerotypeFinder v2.0.1 (2020-07-27; Database v1.0.0, 2022-05-16) and ECTyper v1.0.0rc1 (Database v1.0), both executed with default parameters.

## Supplementary Information

Below is the link to the electronic supplementary material.

Supplementary Material 1

Supplementary Material 2

## Data Availability

The primary genomic sequences and metadata analyzed in this study were retrieved from the publicly available EnteroBase database. The final filtered dataset, consisting of 11,272 Escherichia coli genome assemblies along with their corresponding O: H serotype annotations, is documented in the Supplementary Information (Table S1). Any additional data supporting the study’s findings are available from the corresponding author upon reasonable request.
