# Supplementary figures and images for: Data-driven classification of Escherichia coli using protein language model ascertains O-serotype determining genes
Source: Sci Rep. 2026 Mar 19;16:14232. doi: 10.1038/s41598-026-40783-1 (PMC13139461; doi:10.1038/s41598-026-40783-1)

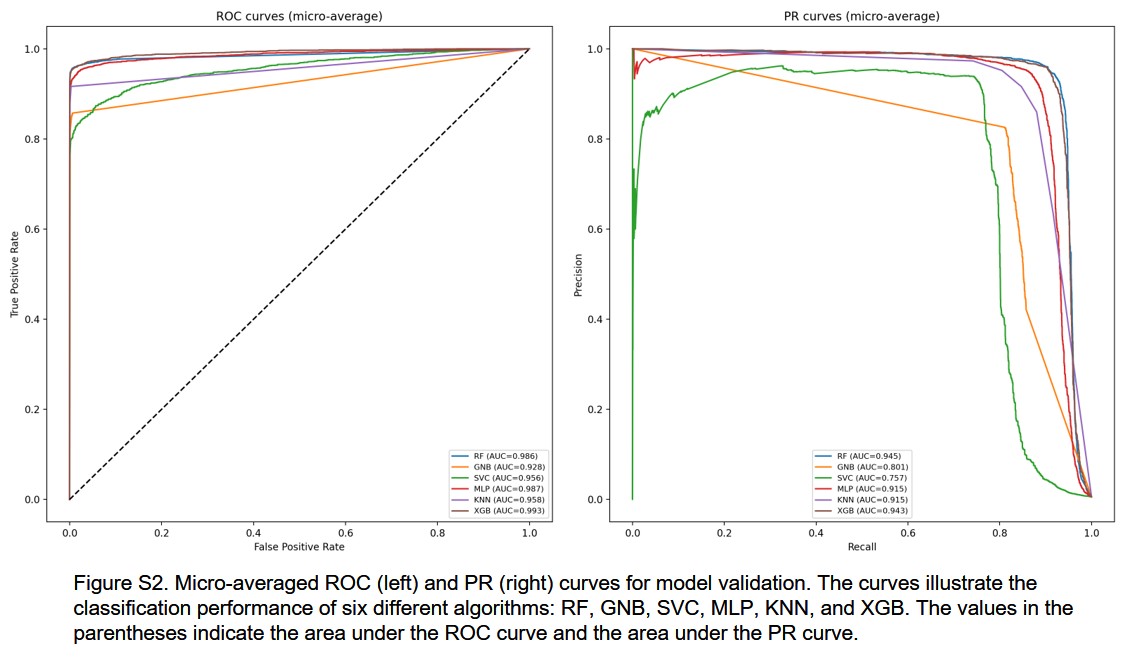

Supplement: Supplementary file 1 — Supplementary Material 1 [file 41598_2026_40783_MOESM1_ESM.jpg]
